# Triggering of the dsRNA Sensors TLR3, MDA5, and RIG-I Induces CD55 Expression in Synovial Fibroblasts

**DOI:** 10.1371/journal.pone.0035606

**Published:** 2012-05-10

**Authors:** Olga N. Karpus, Kirstin M. Heutinck, Paul J. M. Wijnker, Paul P. Tak, Jörg Hamann

**Affiliations:** 1 Department of Experimental Immunology, Academic Medical Center, University of Amsterdam, Amsterdam, The Netherlands; 2 Renal Transplant Unit, Academic Medical Center, University of Amsterdam, Amsterdam, The Netherlands; 3 Division of Clinical Immunology and Rheumatology, Department of Internal Medicine, Academic Medical Center, University of Amsterdam, Amsterdam, The Netherlands; Institut Jacques Monod, France

## Abstract

**Background:**

CD55 (decay-accelerating factor) is a complement-regulatory protein highly expressed on fibroblast-like synoviocytes (FLS). CD55 is also a ligand for CD97, an adhesion-type G protein-coupled receptor abundantly present on leukocytes. Little is known regarding the regulation of CD55 expression in FLS.

**Methods:**

FLS isolated from arthritis patients were stimulated with pro-inflammatory cytokines and Toll-like receptor (TLR) ligands. Transfection with polyinosinic-polycytidylic acid (poly(I:C)) and 5′-triphosphate RNA were used to activate the cytoplasmic double-stranded (ds)RNA sensors melanoma differentiation-associated gene 5 (MDA5) and retinoic acid-inducible gene-I (RIG-I). CD55 expression, cell viability, and binding of CD97-loaded beads were quantified by flow cytometry.

**Results:**

CD55 was expressed at equal levels on FLS isolated from patients with rheumatoid arthritis (RA), osteoarthritis, psoriatic arthritis and spondyloarthritis. CD55 expression in RA FLS was significantly induced by IL-1β and especially by the TLR3 ligand poly(I:C). Activation of MDA5 and RIG-I also enhanced CD55 expression. Notably, activation of MDA5 dose-dependently induced cell death, while triggering of TLR3 or RIG-I had a minor effect on viability. Upregulation of CD55 enhanced the binding capacity of FLS to CD97-loaded beads, which could be blocked by antibodies against CD55.

**Conclusions:**

Activation of dsRNA sensors enhances the expression of CD55 in cultured FLS, which increases the binding to CD97. Our findings suggest that dsRNA promotes the interaction between FLS and CD97-expressing leukocytes.

## Introduction

Rheumatoid arthritis (RA) is a chronic inflammatory autoimmune disease of the joints that is characterized by a marked thickening of the synovium due to neovascularization, fibroblast proliferation, and the recruitment of macrophages and other immune cells [1]. The local production of enzymes and cytokines, and the activation of osteoclasts cause cartilage degradation and bone erosion, finally leading to joint destruction and functional disability.

Fibroblast-like synoviocytes (FLS) are unique cells of mesenchymal origin that constitute the intimal lining, which comprises 2–3 cell layers in normal conditions but can increase up to 15 layers in RA [2]–[4]. Due to the border position between synovial tissue and synovial fluid, FLS obtain signals from both compartments and affect synovial tissue homeostasis in many ways. Moreover, it is increasingly appreciated that FLS contribute to the pathogenesis of RA by regulating inflammatory processes and, more directly, by eroding cartilage. A cell surface marker that defines FLS is CD55. The presence of CD55 in the intimal lining was initially reported by Medof et al. [5]. Later work by Stevens et al. and Edwards and Wilkinson identified CD55 as a marker with an apparent specificity for intimal fibroblasts in synovial disease [6], [7].

CD55, also known as decay-accelerating factor (DAF), is a broadly expressed cell surface molecule that protects cells from self-inflicted damage mediated by complement activation. CD55 controls complement by accelerating the decay of C3/C5 convertases [8]. In line with this well-established function, CD55-deficient mice develop increased complement-mediated autoimmunity in a variety of antibody-driven models [9]. Next to its role as a complement regulator, CD55 is a binding partner of CD97, an adhesion-type G protein-coupled receptor (GPCR) abundantly expressed on almost all leukocytes [10]–[13]. Adhesion-GPCRs are nonclassical heptahelical receptors that facilitate cell and matrix interactions of various cell types [14]. CD97-positive macrophages closely associate with CD55-expressing FLS in the synovial intima [15]. Using CD97-specific multivalent fluorescent probes, we previously demonstrated the ability of CD97 to interact with CD55 on FLS in RA synovium [16]. Based on the site-specific expression of CD55 and CD97, and the finding that CD97 facilitates leukocyte adhesion *in vitro* [11], we postulated that the interaction of CD97^+^ intimal macrophages with CD55^+^ FLS might facilitate the accumulation of inflammatory cells in the synovial tissue of RA patients. Using gene-deficient mice, we recently demonstrated that lack of both CD55 and CD97 indeed ameliorates disease activity in collagen-induced and K/BxN serum transfer models of RA [17].

**Table 1:** Clinical features and treatment of RA, OA, PsA, and SpA patients included in the study.

	RA patients n = 12	OA patients n = 5	PsA patients n = 4	SpA patients n = 5
Sex, no. males/females	2/10	2/3	4/0	1/4
Age, years median (range)	62 (49–80)	63 (50–67)	41 (36–54)	41 (33–57)
DoD, months median (range)	90 (2–414)	24 (12–100)	85 (2–180)	288 (0–384)
IgM-RF, no. positive/negative	9/3	n/a	n/a	5/0
ACPA, no. positive/negative	11/1	n/a	n/a	5/0
No medication, no.	0	3	2	2
NSAIDs, no.	8	2	2	0
MTX, no.	11	0	0	3
DMARDs, no.	7	0	0	2

No  =  number of patients; DoD  =  duration of disease; IgM-RF  =  immunoglobulin M-rheumatoid factor; ACPA  =  anti-citrullinated peptide antibodies; MTX  =  Methotrexate; NSAIDs  =  non-steroidal anti-inflammatory drugs; DMARDs: disease-modifying anti-rheumatic drugs; n/a  =  not available.

The high and cell type-specific expression of CD55 raises the question how CD55 expression is triggered in FLS. FLS can be activated by cytokines and molecular patterns, originating from damaged cells, present in the synovial fluid [2]–[4]. We therefore tested the ability of a range of pro-inflammatory cytokines and Toll-like receptor (TLR) ligands to induce CD55 expression in cultured FLS. We show that CD55 was strongly upregulated by triggering of TLR3, an endosomal pattern recognition receptor involved in the detection of double-stranded (ds)RNA. Stimulation of the cytoplasmic dsRNA sensors melanoma differentiation-associated gene 5 (MDA5) and retinoic acid-inducible gene-I (RIG-I) induced CD55 expression as well. Notably, activation of MDA5, but hardly TLR3 or RIG-I triggering, caused cell death in cultured FLS. Finally, we show that TLR3 activation enhanced the binding of CD97-loaded beads in FLS in a CD55-dependent manner, suggesting that dsRNA increases the interaction of FLS with CD97-positive leukocytes.

## Materials and Methods

### Isolation and Culture of FLS and Dermal Fibroblasts

Synovial tissue samples were obtained by needle arthroscopy from patients with different forms of arthritis [18]. RA patients fulfilled the American College of Rheumatology (ACR) criteria [19], non-psoriatic spondylarthritis (SpA) patients fulfilled European Spondylarthropathy Study Group (ESSG) criteria [20], patients with psoriatic arthritis (PsA) fulfilled the Classification Criteria of Psoriatic Arthritis (CASPAR) study group criteria [21], and patients with inflammatory osteoarthritis (OA) fulfilled established criteria [22] and had a joint effusion in the absence of rheumatic disease other than OA. Clinical data on patients and medication are presented in Table 1. The study was approved by the Medical Ethics Committee of the Academic Medical Center, and all patients gave written informed consent.

**Figure 1:**
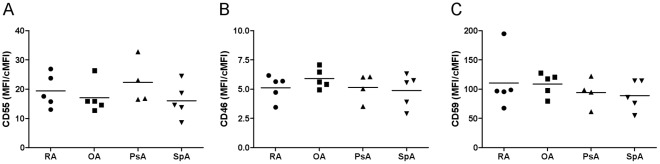
Expression of complement regulatory proteins on cultured FLS of patients with different forms of arthritis. CD55, CD46, and CD59 expression was measured by flow cytometry on cultured FLS from patients with RA, OA, PsA, and SpA. Indicated is the fold difference in mean fluorescence intensity (MFI) over respective isotype control Ig (cMFI) (mean, n = 4−5).

Single cell suspensions were generated by finely mincing freshly isolated synovial tissue, followed by treatment with 0.5 mg/ml collagenase type VIII (Sigma-Aldrich, Zwijndrecht, The Netherlands) for 2 h at 37°C. The obtained cell suspension was cultured in Dulbecco’s Eagle’s medium (DMEM; 1 g/l D-glucose) supplemented with 10% heat inactivated fetal calf serum (FCS), L-glutamine, HEPES, and antibiotics (penicillin, gentamicin, and streptomycin) (Gibco, Breda, The Netherlands). Non-adherent cells were removed after 24 h, and adhering cells were grown to sub-confluence (80%) and subsequently split (1∶3) by trypsinization. Synovial fibroblasts were used for experiments from passage 3 until passage 9; at that time cultures were free of macrophages and non-fibroblasts.

Primary dermal fibroblasts, obtained from biopsy samples of normal skin, were kindly provided by Dr. Marcel Teunissen (AMC, Amsterdam, The Netherlands). The cells were cultured in Ham’s F-12 medium (Gibco) with 10% FCS and used for experiments between passage 3 and 5.

**Figure 2:**
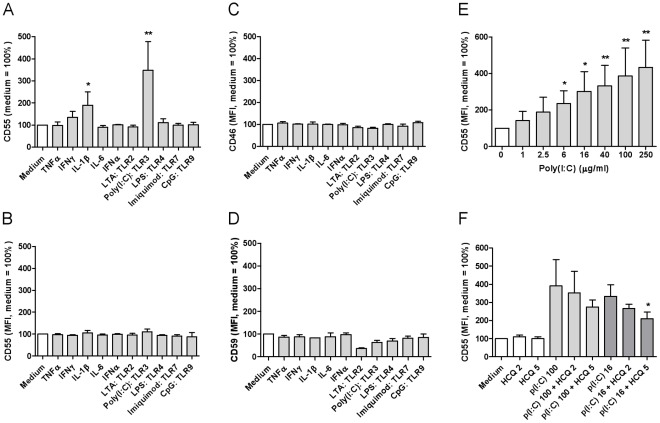
CD55 is upregulated by poly(I:C) and IL-1β on synovial fibroblasts. RA-derived synovial fibroblasts (**A, C-F**) and dermal fibroblasts (**B**) were starved overnight and subsequently stimulated for 2 days with 100 ng/ml TNFα, 100 ng/ml IFNγ, 100 ng/ml IL-1β, 1 ng/ml IL-6, 100 U/ml IFNα, 100 µg/ml LTA (TLR2 ligand), 100 µg/ml poly(I:C) (TLR3 ligand), 10 µg/ml LPS (TLR4 ligand), 100 µg/ml imiquimod (TLR7 ligand), or 10 µg/ml CpG oligonucleotides (TLR9 ligand). Expression of CD55 (**A** and **B**), CD46 (**C**) and CD59 (**D**) was studied by flow cytometry. **E,** Upregulation of CD55 in response to increasing concentrations of poly(I:C). **F,** Inhibition of CD55 upregulation by chloroquine (HCQ), an inhibitor of endosomal acidification, added prior to poly(I:C) stimulation. Indicated is the relative protein expression as percentage of the medium control (mean ± SD, n = 6 (**A**) and 3–5 (**B-F**)). *, p<0.05; **, p<0.005.

### Reagents and Stimulation Assays

Synovial and dermal fibroblasts were cultured in 6-well plates and allowed to grow to 70–90% confluence. After serum starvation over night in DMEM containing 1% FCS, the cells were stimulated for 48 h with the following agents: tumor necrosis factor α (TNFα; 100 ng/ml; BioSource International, Camarillo, CA, USA), interferon α (IFNα2a; 100 or 1000 U/ml; PBL Biomedical Laboratories, Piscataway, NJ, USA), IFNβ (100 or 1000 U/ml; Peprotech, Rocky Hill, NJ, USA), interleukin 1β (IL-1β; 100 ng/ml), IL-6 (1 ng/ml) (both Invitrogen, Breda, The Netherlands), IFNγ (100 ng/ml), lipoteichoic acid from *Staphylococcus aureus* (LTA; 100 µg/ml), polyinosinic-polycytidylic acid (poly(I:C); from 0.01–250 µg/ml), lipopolysaccharide from *Escherichia coli* K-235 (LPS; 10 µg/ml), imiquimod (100 µg/ml) (all Sigma-Aldrich), and CpG oligonucleotides (10 µg/ml; Invivogen, San Diego, CA, USA). When indicated, hydroxychloroquine (HCQ; 2–5 µg/ml; Sigma-Aldrich) was added to the cultures 2 h prior to stimulation with poly(I:C). For intracellular delivery of poly(I:C) and 5′-triphosphate RNA (3pRNA; kindly provided by Prof. G. Hartmann and Dr. M. Schlee, University Hospital Bonn, Germany) transfection reagent Fugene HD (Roche, Mannheim, Germany) was used according to the manufacturer’s protocol.

### Flow Cytometry

For measurement of CD55, CD46, and CD59 surface expression, FLS were detached from 12-well plates with TrypLE™ (Gibco), washed with PBS/0.5% bovine serum albumine (BSA), and incubated for 30 min at 4°C with the following monoclonal antibodies: CD55-APC (1∶50; BD Biosciences, Franklin Lakes, NJ), CD46-FITC (1∶50; AbD Serotec; Raleigh, NC, USA), and CD59-PE (1∶100; eBiosciences, San Diego, CA, USA) or isotype control antibodies: IgG2a-APC (1∶50), IgG1-FITC (1∶50), and IgG2a-PE (1∶100) (all BD, Breda, The Netherlands).

To study the expression and accessibility of particular short consensus repeats (SCR) of CD55, cells were incubated with monoclonal antibodies LA1 (anti-SCR1), LA2 (anti-SCR3), LA4 (anti-SCR4), LA5 (anti-stalk) (all kindly provided by Prof. Lucien Aarden, Sanquin Research, Amsterdam, The Netherlands), and BRIC110 (anti-SCR2; IBGRL, Filton, Bristol, UK) or with control mouse IgG (all 5 µg/ml). After washing, cells were incubated with APC-labeled goat-anti-mouse antibody (1∶300; BD Biosciences).

To quantify cell death, cells were incubated with Annexin-V-FITC (1∶100; IQ Products, Groningen, The Netherlands) for 30 min at 4°C in calcium buffer. Before measurement, propidium iodide (5 ng/ml; Sigma) was added.

All stainings were visualized by flow cytometry on a FACSCalibur (Becton Dickinson, San Jose, CA, USA), and results were analyzed using the FlowJo software package (Tree Stars, Ashland, OR, USA).

**Figure 3:**
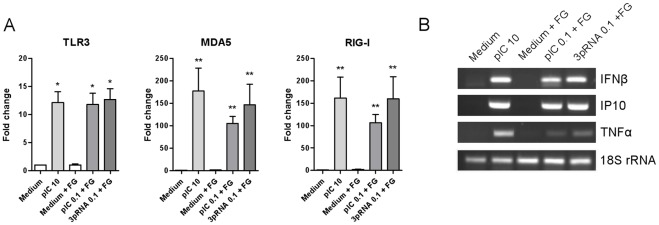
FLS express functional cytoplasmic dsRNA sensors. RA-derived synovial fibroblasts were stimulated for 16 h with the indicated concentrations (µg/ml) of poly(I:C), poly(I:C) with fugene, or 3pRNA with fugene to trigger, respectively, TLR3, MDA5, and RIG-I. Transcription levels of (**A**) TLR3, MDA5, and RIG-I, and (**B**) the anti-viral/pro-inflammatory response genes IFN β, IP-10, and TNFα was measured by quantitative and semiquantitative PCR, respectively. Depicted is the fold change gene expression compared to medium control (mean ± SD, n = 4) (**A**) or representative photographs (**B**). *, p<0.05, **, p<0.005.

**Figure 4:**
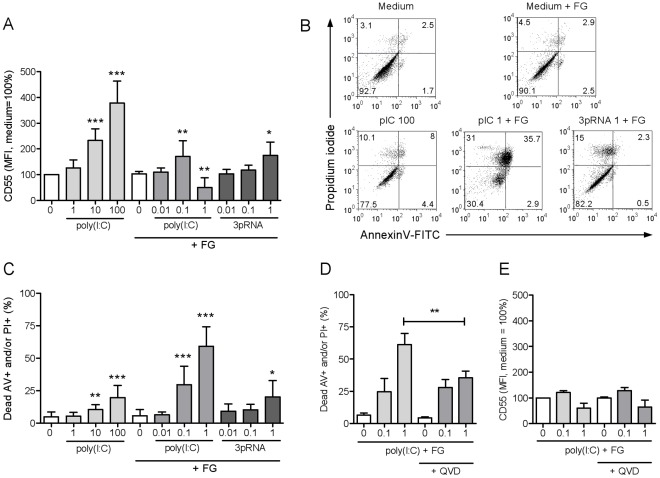
Stimulation of cytoplasmic dsRNA receptors in FLS upregulates CD55 expression and, through MDA5, induces cell death. RA-derived synovial fibroblasts were stimulated for 2 days with the indicated concentrations (µg/ml) of poly(I:C), poly(I:C) with fugene, or 3pRNA with fugene to trigger, respectively, TLR3, MDA5, and RIG-I. **A,** Expression of CD55 analyzed by flow cytometry (mean ± SD, n = 6). **B,** Representative flow cytometry plots of annexin V and propidium iodide staining. **C,** Percentages of annexin V and/or propidium iodide-positive cells analyzed by flow cytometry (mean ± SD, n = 6). **D, E,** Effect of the pan-caspase inhibitor QVD on cell death and CD55 expression induced by intracellular delivery of poly(I:C) (mean ± SD, n = 3). *, p<0.05; **, p<0.005; ***, p<0.001.

### Quantitative and Semi-quantitative PCR

FLS were detached from 6-wells plates as described above, and RNA was isolated using the Invisorb spin cell RNA mini kit (Westburg, Leusden, The Netherlands). RNA quantity and purity was measured on a NanoDrop (ND-1000; NanoDrop Technologies, Rockland, DE, USA). Reverse transcription was performed with random hexamer primer and SuperScript II RNase H-reverse transcriptase kit (Invitrogen) according to manufacturer’s protocol. Transcript levels of dsRNA sensors (TLR3, MDA5, RIG-I) were analyzed by quantitative PCR with the StepOnePlus Real-Time PCR system using Fast SYBR® Green Master Mix (Applied Biosystems, Carlsbad, CA, USA). Gene transcription was normalized to 18S rRNA (ΔCt). The relative expression ratios were calculated using the 2^−ΔΔCt^ method. Transcript levels of cytokines (IFNβ, IP10, TNFα) were analyzed by semi-quantitative PCR using Salsa polymerase (MRC Holland, Amsterdam, The Netherlands) and the Bio-Rad C1000 Thermal cycler (Bio-Rad laboratories, Veenendaal, The Netherlands). PCR products were visualized in agarose gels. Primer sequences and annealing temperatures for all PCRs are depicted in Table S1.

**Figure 5:**
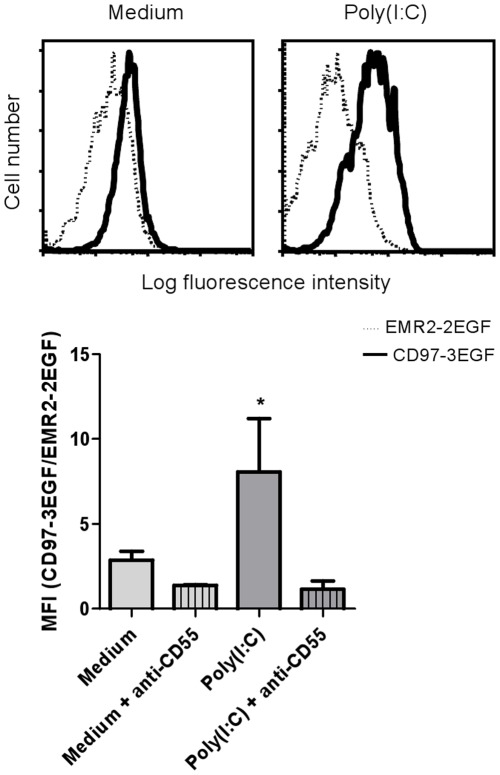
Poly(I:C)-induced upregulation of CD55 on synovial fibroblasts increases the binding capacity for CD97. Synovial fibroblasts were stimulated for 2 days with 100 µg/ml poly(I:C). Affinity for CD97 was measured with multivalent fluorescent probes loaded with recombinant CD97-3EGF or EMR2-2EGF (control). To confirm specificity, cells were preincubated with mAb CLB-CD97L/1, directed against the first SCR of CD55. On top, representative histogram plots are shown. The bars represent the fold difference in mean fluorescence intensity (MFI) for CD97-3EGF over EMR2-2EGF (mean ± SD, n = 3). *, p<0.05.

### CD97-binding Assay

Cell-binding assays using biotinylated Fc-proteins coupled to fluorescent beads were performed as described previously [23]. Briefly, 10 µl avidin-coated fluorescent beads (Spherotech, Libertyville, IL, USA) were washed and incubated with saturating amounts (>1 µg) of biotinylated recombinant protein (CD97-3EGF or EMR2-2EGF; kindly provided by Organon-Schering Plough, Cambridge, MA). After 1 h, non-binding protein was removed, and the bead-protein complexes were sonicated immediately before addition to poly(I:C)-stimulated or unstimulated FLS for 1 h. For blocking studies, cells were preincubated for 30 min with CLB-CD97L/1 ascitis (1∶2000, anti-CD55). Adherence of beads to the cells was analyzed by flow cytometry (FACSCalibur).

### Statistical Analysis

Statistical analyses were performed in SPSS (version 16.0; SPSS, Chicago, IL, USA) and Graph Pad Prism (version 5; GraphPad software, La Jolla, CA, USA). Protein expression, mRNA levels and amount of apoptotic cells on stimulated synovial fibroblasts were compared to unstimulated cells with two-tailed paired T-test. Expression on synovial fibroblasts of different arthritides was compared using two-tailed Mann Whitney U tests. A two-tailed unpaired T-test was used to compare the levels of fluorescent bead binding.

## Results

### Cultured FLS Express the Complement Regulators CD55, CD46, and CD59

We studied the expression levels of CD55 on FLS from patients with different forms of arthritis by flow-cytometric analysis. CD55 expression levels did not differ between cells from RA, OA, PsA, or SpA (Figure 1A). We also analyzed the expression levels of CD46 and CD59, two other established complement regulators, but found no differences between FLS of different arthritides (Figure 1B and C).

### Poly(I:C) Induces CD55 Expression on FLS through TLR3

To address the regulation of CD55 in FLS, we stimulated cells with various inflammatory cytokines and TLR ligands. IL-1β and especially poly(I:C) significantly enhanced CD55 expression in FLS from RA (Figure 2A, Figure S1) and OA (data not shown). In contrast, CD55 was not upregulated in dermal fibroblasts by any of the tested stimuli (Figure 2B). Moreover, we did not observe induction of CD46 and CD59 in stimulated RA FLS (Figure 2C and D), suggesting specific regulation of CD55 expression in FLS.

Poly(I:C) is an analog for dsRNA of viral origin that dose-dependently induced expression of CD55 on protein (Figure 2E) and mRNA level (data not shown). A specific sensor for poly(I:C) expressed in the endosomal compartment is TLR3 [2], [24]. Chloroquine (HCQ) inhibits endosomal acidification, thereby preventing TLR3 signaling. We found that chloroquine reduced the upregulation of CD55 (Figure 2F), implying that poly(I:C)-induced CD55 expression was mediated by TLR3. Complete inhibition was not accomplished, since the inhibitor was toxic to cultured FLS at concentrations above 5 µg/ml (data not shown).

TLR3 is known to mediate the production of type I IFNs, which subsequently, upon binding to the IFNα/â receptor, can turn on the transcription of a large set of interferon-stimulated genes (ISGs). To test whether type I IFNs regulate CD55 expression, we stimulated FLS with IFNα and IFNβ. Even at high concentrations of 1000 U/ml, expression levels of CD55 were hardly affected (data not shown), indicating that signaling through type I IFN receptors is not sufficient to induce CD55 expression in RA-FLS.

### Stimulation of the Cytoplasmic dsRNA Receptors MDA5 and RIG-I in FLS Causes CD55 Upregulation and, through MDA5, Cell Death

In addition to TLR3, most cell types express the cytoplasmic helicases MDA5 and RIG-I, which sense dsRNA formed during viral replication in infected cells [24]. Activation of MDA5 and RIG-I can be accomplished *in vitro* by transfection of the dsRNA analogs poly(I:C) and 3pRNA, respectively. The expression of TLR3, MDA5, and RIG-I was analyzed in FLS stimulated with these dsRNA mimics, since it is known that receptors expression can be induced by their ligands [25]–[27]. Figure 3A shows that both extra- and intracellular stimulation with poly(I:C) and intracellular stimulation with 3pRNA upregulated transcription of TLR3, MDA5, and RIG-I. Moreover, activation with dsRNA induced transcription of the anti-viral and pro-inflammatory response genes IFNβ, IP-10 (CXCL10), and TNFα (Figure 3B). Thus, FLS are equipped with functional endosomal and cytoplasmic dsRNA sensors.

Next, we tested whether triggering of MDA5 and RIG-I would promote CD55 expression, like TLR3 signaling does. Protein levels of CD55 were indeed elevated in FLS after stimulation of MDA5 or RIG-I (Figure 4A). Notably, transfection with poly(I:C) already at the intermediate concentration of 0.1 µg/ml induced significant cell death. After transfection of 1 µg/ml poly(I:C), about 60% of cells died, whereas high concentrations of extracellular poly(I:C) and 3pRNA caused death in up to 20% of cells (Figure 4B and C). Treatment with the pan-caspase inhibitor QVD reduced cell death at a concentration of poly(I:C) of 1 µg/ml, but had no effect at 0.1 µg/ml, indicating that MDA5-induced cell death in cultured FLS is partially caspase-dependent (Figure 4D). CD55 induction was not influenced by QVD treatment (Figure 4E). We concluded that activation of cytoplasmic dsRNA receptors not only promoted the expression of CD55, but MDA5 signaling also caused cell death in cultured FLS.

### Upregulation of CD55 on FLS Increases the Binding Capacity for CD97

CD55 interacts with CD97 by binding of the SCRs 1, 2, and 3 of the adhesion-GPCR [11], [13]. To study whether synovial fibroblasts of arthritis patients express the CD55 domains involved in CD97 binding, we stained the cells with antibodies recognizing individual SCRs. Flow-cytometric analysis confirmed that FLS of RA, OA, SpA, and PsA patients express full-length CD55 possessing all four SCRs (Figure S2).

We then tested whether induction of CD55 on RA-derived FLS enhanced their binding capacity for CD97 by incubating the cells with fluorescent CD97-loaded beads. Indeed, we observed a significant increase in binding of CD97-loaded beads after TLR3-mediated CD55 upregulation, which was blocked entirely by antibodies against CD55 (Figure 5).

## Discussion

We here show that cultured FLS derived from RA patients express CD55 and that the amount of membrane-bound CD55 is increased by the triggering of dsRNA sensors in these cells. We demonstrate that CD55 induction enhanced the binding of CD97-loaded beads to RA-derived FLS, suggesting that dsRNA promotes the interaction of FLS with CD97-bearing leukocytes. Finally, we report that stimulation of the cytoplasmic dsRNA receptor MDA5 initiates cell death in cultured FLS.

CD55 is an established histological marker for FLS in the intimal lining layer of the synovium in arthritis patients [3], [15]. We previously demonstrated that immunohistochemical staining for CD55 in the intimal lining layer tends to be higher in RA as compared to OA [15]. Currently, it is not known how CD55 expression is regulated in FLS. In other cell types, expression of CD55 is enhanced by cytokines such as TNFα [28], [29], which are highly present in the synovium of RA patients [30]. We observed that in cultured RA FLS, CD55 was not induced by TNFα, yet the pro-inflammatory cytokine IL-1β enhanced its expression by about two-fold. Others have demonstrated that IL-1β also promotes CD55 expression in articular chondrocytes, hepatoma cells, and intestinal epithelial cells [31]–[33]. In contrast to some of these cell types [31], [33], expression of two other complement regulators (CD46 and CD59) was not enhanced by pro-inflammatory cytokines in cultured RA FLS. Furthermore, we observed that none of the tested cytokines or TLR ligands enhanced CD55 expression in dermal fibroblasts, which express functional TLRs [34]. Thus, selective regulation of CD55 expression in FLS is specific for this cell type.

Activation of the dsRNA sensors TLR3, MDA5, and RIG-I robustly enhanced the expression of CD55 in FLS in a dose-dependent manner. Previous studies have shown that FLS express TLR3, MDA5, and RIG-I [35]–[39]. Moreover, these studies demonstrated that TLR3 and RIG-I triggering induces the expression of type I IFNs, cytokines, chemokines, and matrix metalloproteinases and proved activation of the transcription factors activator protein 1 (AP-1), NF-κB, and interferon-regulating factor (IRF) 3 and 7, which are crucially involved in dsRNA sensor-mediated gene expression [24]. We here confirm and extend these findings, and show that also MDA-5 is functional in cultured FLS. In agreement with the well-known ability of dsRNA sensors to induce their own expression [25]–[27], we found that transcription levels of TLR3, MDA5, and RIG-I in cultured RA FLS were upregulated in response to any form of stimulation with dsRNAs. Moreover, triggering of TLR3, MDA5, and RIG-I induced the expression of IFNβ, IP-10, TNFα, and, as shown here, CD55, further demonstrating the functionality of these signaling paths in synovial fibroblasts. The inability of pro-inflammatory cytokines and synovial fluid (data not shown) to upregulate CD55 suggests that CD55 expression is regulated rather by IRF3 and/or IRF7 than by NF-κB or AP-1. IRFs are known to induce the production of type I IFNs, which in turn can trigger interferon-stimulated genes (ISGs) after binding to the IFNα/β receptor. However, IFNα and IFNβ did not induce CD55 expression, indicating that dsRNA-induced CD55 upregulation is not the result of a type I IFN feed-forward loop.

An unexpected finding of this study was the observation that intracellularly delivered poly(I:C) initiates cell death in cultured FLS. Recent studies have shown that dsRNA receptor activation can promote apoptosis in malignant cells [40]–[43]. In many non-malignant cells, dsRNA is not inducing cell death directly but can activate a pro-apoptotic program that renders cells susceptible to other apoptotic stimuli [42]–[45]. We observed that in cultured RA FLS, activation of MDA5 directly induced cell death, which was only partly caspase-dependent. It is known that triggering of MDA5 can induce expression of the pro-apoptotic BH3-only protein Noxa, leading to activation of the mitochondrial apoptosis pathway [27], [42], [45], [46]. Notably, another pro-apoptotic BH3-only protein, Puma, has been identified as a rapid and potent inducer of apoptosis in FLS [47] and can be induced by dsRNA sensor activation as well [27], [42], [45]. Others found that MDA5 triggering induces autophagy and death in melanoma cells [44]. Whether the same mechanisms play a role in FLS remains to be identified. In addition, it needs to be investigated whether the initiation of cell death upon MDA5 triggering relates to the neoplastic phenotype that has been described for FLS in RA [3]. Beyond the initial scope of our study, the observation that triggering of MDA5 effectively induces death in synovial fibroblasts, raises the possibility that MDA5 might be a therapeutic target for the reduction of intimal hyperplasia in RA.

An important question relates to the possible source of the stimuli that trigger CD55 expression in cultured FLS. IL-1β is a critical cytokine in autoinflammatory diseases that is released upon caspase-1-dependent and independent processing [48]. Moreover, both IL-1β and dsRNA become available during viral infection [24]. While dsRNA represents the genome of some viruses and is an intermediate formed by many viruses during their replication, the role of viruses in the etiology and pathology of RA is uncertain [49]–[51]. Next to viral dsRNA, TLR3 has been shown to recognize endogenous RNA released from necrotic cells [35], [52]–[54]; however, our attempts to induce CD55 with either synovial fluid or necrotic FLS failed (data not shown). Interestingly, extracellular RNA is abundantly present in the synovial lining [55], and it remains to be shown whether chemical modifications that protect endogenous RNAs from enzymatic degradation can generate *de novo* ligands that are able trigger dsRNA sensors in FLS. In line herewith, recent evidence suggests that RIG-I-like helicases are modifiers of sterile inflammation in the mouse colon and brain [56], [57].

Together, our data indicate that inflammatory and viral cues in synovial tissue may upregulate CD55 on FLS, which can facilitate the interaction with CD97^+^ leukocytes. As growing evidence suggests that CD55 is part of the stromal address code that directs the retention and survival of inflammatory immune cells in the synovium [16], [58], it will be important to study the expression of CD55 in early arthritis and in various forms of established arthritis.

## Supporting Information

Figure S1 **CD55 is upregulated by IL-1β and poly(I:C) on synovial fibroblasts.** RA derived synovial fibroblasts were stimulated for two days with 100 ng/ml IL1β or 100 µg/ml poly(I:C). CD55 expression on synovial fibroblasts was analysed by flow cytometry. Representative scatter plot and histograms are shown.
(TIF)

Figure S2 **Synovial fibroblasts express full-length CD55.** Synovial fibroblasts of rheumatoid arthritis (RA), osteoarthritis (OA), psoriatic arthritis (PsA), and spondyloarthritis (SpA) patients were analyzed by flow cytometry with domain-specific antibodies recognizing either the stalk or individual short consensus repeats (SCR) 1 to 4 and the stalk region of CD55. Representative histograms of 3 experiments are shown.
(TIF)

Table S1 **PCR primer specification.**
(TIF)

## References

[1] Tak PP, Bresnihan B (2000). The pathogenesis and prevention of joint damage in rheumatoid arthritis: advances from synovial biopsy and tissue analysis.. Arthritis Rheum.

[2] Noss EH, Brenner MB (2008). The role and therapeutic implications of fibroblast-like synoviocytes in inflammation and cartilage erosion in rheumatoid arthritis.. Immunol Rev.

[3] Bartok B, Firestein GS (2010). Fibroblast-like synoviocytes: key effector cells in rheumatoid arthritis.. Immunol Rev.

[4] Neumann E, Lefevre S, Zimmermann B, Gay S, Muller-Ladner U (2010). Rheumatoid arthritis progression mediated by activated synovial fibroblasts.. Trends Mol Med.

[5] Medof ME, Walter EI, Rutgers JL, Knowles DM, Nussenzweig V (1987). Identification of the complement decay-accelerating factor (DAF) on epithelium and glandular cells and in body fluids.. J Exp Med.

[6] Stevens CR, Mapp PI, Revell PA (1990). A monoclonal antibody (Mab 67) marks type B synoviocytes.. Rheumatol Int.

[7] Edwards JC, Wilkinson LS (1996). Distribution in human tissues of the synovial lining-associated epitope recognised by monoclonal antibody 67.. J Anat.

[8] Lublin DM, Atkinson JP (1989). Decay-accelerating factor: biochemistry, molecular biology, and function.. Annu Rev Immunol.

[9] Song WC (2006). Complement regulatory proteins and autoimmunity.. Autoimmunity.

[10] Hamann J, Eichler W, Hamann D, Kerstens HM, Poddighe PJ (1995). Expression cloning and chromosomal mapping of the leukocyte activation antigen CD97, a new seven-span transmembrane molecule of the secretion receptor superfamily with an unusual extracellular domain.. J Immunol.

[11] Hamann J, Vogel B, van Schijndel GMW, van Lier RAW (1996). The seven-span transmembrane receptor CD97 has a cellular ligand (CD55, DAF).. J Exp Med.

[12] Eichler W, Hamann J, Aust G (1997). Expression characteristics of the human CD97 antigen.. Tissue Antigens.

[13] Lin HH, Stacey M, Saxby C, Knott V, Chaudhry Y (2001). Molecular analysis of the epidermal growth factor-like short consensus repeat domain-mediated protein-protein interactions: dissection of the CD97-CD55 complex.. J Biol Chem.

[14] Yona S, Lin HH, Siu WO, Gordon S, Stacey M (2008). Adhesion-GPCRs: emerging roles for novel receptors.. Trends Biochem Sci.

[15] Hamann J, Wishaupt JO, van Lier RA, Smeets TJ, Breedveld FC (1999). Expression of the activation antigen CD97 and its ligand CD55 in rheumatoid synovial tissue.. Arthritis Rheum.

[16] Kop EN, Kwakkenbos MJ, Teske GJ, Kraan MC, Smeets TJ (2005). Identification of the epidermal growth factor-TM7 receptor EMR2 and its ligand dermatan sulfate in rheumatoid synovial tissue.. Arthritis Rheum.

[17] Hoek RM, de Launay D, Kop EN, Yilmaz-Elis AS, Lin F (2010). Deletion of either CD55 or CD97 ameliorates arthritis in mouse models.. Arthritis Rheum.

[18] Gerlag DM, Tak PP (2009). How to perform and analyse synovial biopsies.. Best Pract Res Clin Rheumatol.

[19] Arnett FC, Edworthy SM, Bloch DA, McShane DJ, Fries JF (1988). The American Rheumatism Association 1987 revised criteria for the classification of rheumatoid arthritis.. Arthritis Rheum.

[20] Dougados M, van der Linden S, Juhlin R, Huitfeldt B, Amor B (1991). The European Spondylarthropathy Study Group preliminary criteria for the classification of spondylarthropathy.. Arthritis Rheum.

[21] Taylor W, Gladman D, Helliwell P, Marchesoni A, Mease P (2006). Classification criteria for psoriatic arthritis: development of new criteria from a large international study.. Arthritis Rheum.

[22] Altman R, Asch E, Bloch D, Bole G, Borenstein D (1986). Development of criteria for the classification and reporting of osteoarthritis. Classification of osteoarthritis of the knee. Diagnostic and Therapeutic Criteria Committee of the American Rheumatism Association.. Arthritis Rheum.

[23] Stacey M, Chang GW, Sanos SL, Chittenden LR, Stubbs L (2002). EMR4, a novel epidermal growth factor (EGF)-TM7 molecule up-regulated in activated mouse macrophages, binds to a putative cellular ligand on B lymphoma cell line A20.. J Biol Chem.

[24] Takeuchi O, Akira S (2010). Pattern recognition receptors and inflammation.. Cell.

[25] Imaizumi T, Tanaka H, Matsumiya T, Yoshida H, Tanji K (2010). Retinoic acid-inducible gene-I is induced by double-stranded RNA and regulates the expression of CC chemokine ligand (CCL) 5 in human mesangial cells.. Nephrol Dial Transplant.

[26] Kariko K, Ni H, Capodici J, Lamphier M, Weissman D (2004). mRNA is an endogenous ligand for Toll-like receptor 3.. J Biol Chem.

[27] Heutinck KM, Kassies J, Florquin S, ten Berge IJ, Hamann J (2012). SerpinB9 expression in human renal tubular epithelial cells is induced by triggering of the viral dsRNA sensors TLR3, MDA5 and RIG-I.. Nephrol Dial Transplant.

[28] Halme J, Sachse M, Vogel H, Giese T, Klar E (2009). Primary human hepatocytes are protected against complement by multiple regulators.. Mol Immunol.

[29] Ahmad SR, Lidington EA, Ohta R, Okada N, Robson MG (2003). Decay-accelerating factor induction by tumour necrosis factor-alpha, through a phosphatidylinositol-3 kinase and protein kinase C-dependent pathway, protects murine vascular endothelial cells against complement deposition.. Immunology.

[30] Tak PP, Smeets TJ, Daha MR, Kluin PM, Meijers KA (1997). Analysis of the synovial cell infiltrate in early rheumatoid synovial tissue in relation to local disease activity.. Arthritis Rheum.

[31] Hyc A, Osiecka-Iwan A, Strzelczyk P, Moskalewski S (2003). Effect of IL-1beta, TNF-alpha and IL-4 on complement regulatory protein mRNA expression in human articular chondrocytes.. Int J Mol Med.

[32] Nasu J, Mizuno M, Uesu T, Takeuchi K, Inaba T (1998). Cytokine-stimulated release of decay-accelerating factor (DAF;CD55) from HT-29 human intestinal epithelial cells.. Clin Exp Immunol.

[33] Spiller OB, Criado-Garcia O, Rodriguez De CS, Morgan BP (2000). Cytokine-mediated up-regulation of CD55 and CD59 protects human hepatoma cells from complement attack.. Clin Exp Immunol.

[34] Farina GA, York MR, Di MM, Collins CA, Meller S (2010). Poly(I:C) drives type I IFN- and TGFbeta-mediated inflammation and dermal fibrosis simulating altered gene expression in systemic sclerosis.. J Invest Dermatol.

[35] Brentano F, Schorr O, Gay RE, Gay S, Kyburz D (2005). RNA released from necrotic synovial fluid cells activates rheumatoid arthritis synovial fibroblasts via Toll-like receptor 3.. Arthritis Rheum.

[36] Lundberg AM, Drexler SK, Monaco C, Williams LM, Sacre SM (2007). Key differences in TLR3/poly I:C signaling and cytokine induction by human primary cells: a phenomenon absent from murine cell systems.. Blood.

[37] Ospelt C, Brentano F, Rengel Y, Stanczyk J, Kolling C (2008). Overexpression of toll-like receptors 3 and 4 in synovial tissue from patients with early rheumatoid arthritis: toll-like receptor expression in early and longstanding arthritis.. Arthritis Rheum.

[38] Imaizumi T, Arikawa T, Sato T, Uesato R, Matsumiya T (2008). Involvement of retinoic acid-inducible gene-I in inflammation of rheumatoid fibroblast-like synoviocytes.. Clin Exp Immunol.

[39] Carrion M, Juarranz Y, Perez-Garcia S, Jimeno R, Pablos JL (2011). RNA sensors in human osteoarthritic and rheumatoid synovial fibroblasts. Immune regulation by vasoactive intestinal peptide.. Arthritis Rheum.

[40] Paone A, Starace D, Galli R, Padula F, De Cesaris P (2008). Toll-like receptor 3 triggers apoptosis of human prostate cancer cells through a PKC-alpha-dependent mechanism.. Carcinogenesis.

[41] Peng S, Geng J, Sun R, Tian Z, Wei H (2009). Polyinosinic-polycytidylic acid liposome induces human hepatoma cells apoptosis which correlates to the up-regulation of RIG-I like receptors.. Cancer Sci.

[42] Besch R, Poeck H, Hohenauer T, Senft D, Hacker G (2009). Proapoptotic signaling induced by RIG-I and MDA-5 results in type I interferon-independent apoptosis in human melanoma cells.. J Clin Invest.

[43] Weber A, Kirejczyk Z, Besch R, Potthoff S, Leverkus M (2010). Proapoptotic signalling through Toll-like receptor-3 involves TRIF-dependent activation of caspase-8 and is under the control of inhibitor of apoptosis proteins in melanoma cells.. Cell Death Differ.

[44] Tormo D, Checinska A, Alonso-Curbelo D, Perez-Guijarro E, Canon E (2009). Targeted activation of innate immunity for therapeutic induction of autophagy and apoptosis in melanoma cells.. Cancer Cell.

[45] Heutinck KM, Rowshani AT, Kassies J, Claessen N, Bemelman FJ (2012). The viral dsRNA sensors TLR3, MDA5 and RIG-I incuce anti-viral, pro-inflammatory and pro-apoptotic responses in human renal tubular epithelial cells.. Kidney Int.

[46] Lallemand C, Blanchard B, Palmieri M, Lebon P, May E (2007). Single-stranded RNA viruses inactivate the transcriptional activity of p53 but induce NOXA-dependent apoptosis via post-translational modifications of IRF-1, IRF-3 and CREB.. Oncogene.

[47] Cha HS, Rosengren S, Boyle DL, Firestein GS (2006). PUMA regulation and proapoptotic effects in fibroblast-like synoviocytes.. Arthritis Rheum.

[48] Dinarello CA (2011). Interleukin-1 in the pathogenesis and treatment of inflammatory diseases.. Blood.

[49] Stahl HD, Hubner B, Seidl B, Liebert UG, van der Heijden IM (2000). Detection of multiple viral DNA species in synovial tissue and fluid of patients with early arthritis.. Ann Rheum Dis.

[50] Munz C, Lunemann JD, Getts MT, Miller SD (2009). Antiviral immune responses: triggers of or triggered by autoimmunity?. Nat Rev Immunol.

[51] Becker J, Winthrop KL (2010). Update on rheumatic manifestations of infectious diseases.. Curr Opin Rheumatol.

[52] Kariko K, Ni H, Capodici J, Lamphier M, Weissman D (2004). mRNA is an endogenous ligand for Toll-like receptor 3.. J Biol Chem.

[53] Cavassani KA, Ishii M, Wen H, Schaller MA, Lincoln PM (2008). TLR3 is an endogenous sensor of tissue necrosis during acute inflammatory events.. J Exp Med.

[54] Lai Y, Di Nardo A, Nakatsuji T, Leichtle A, Yang Y (2009). Commensal bacteria regulate Toll-like receptor 3-dependent inflammation after skin injury.. Nat Med.

[55] Zimmermann B, Fischer S, Stürz H, Rehart S, Lehr A (2012). Expression of extracellular RNA in synovial tissue and RNase activity in synovial fluid of rheumatoid arthritis patients.. Ann Rheum Dis.

[56] Wang Y, Zhang HX, Sun YP, Liu ZX, Liu XS (2007). Rig-I−/− mice develop colitis associated with downregulation of G alpha i2.. Cell Res.

[57] Dann A, Poeck H, Croxford AL, Gaupp S, Kierdorf K (2012). Cytosolic RIG-I-like helicases act as negative regulators of sterile inflammation in the CNS.. Nat Neurosci.

[58] Buckley CD (2011). Why does chronic inflammation persist: An unexpected role for fibroblasts.. Immunol Lett.

