# Supplementary figures and images for: Triggering of the dsRNA Sensors TLR3, MDA5, and RIG-I Induces CD55 Expression in Synovial Fibroblasts
Source: PLoS One. 2012 May 10;7(5):e35606. doi: 10.1371/journal.pone.0035606 (PMC3349673; doi:10.1371/journal.pone.0035606)

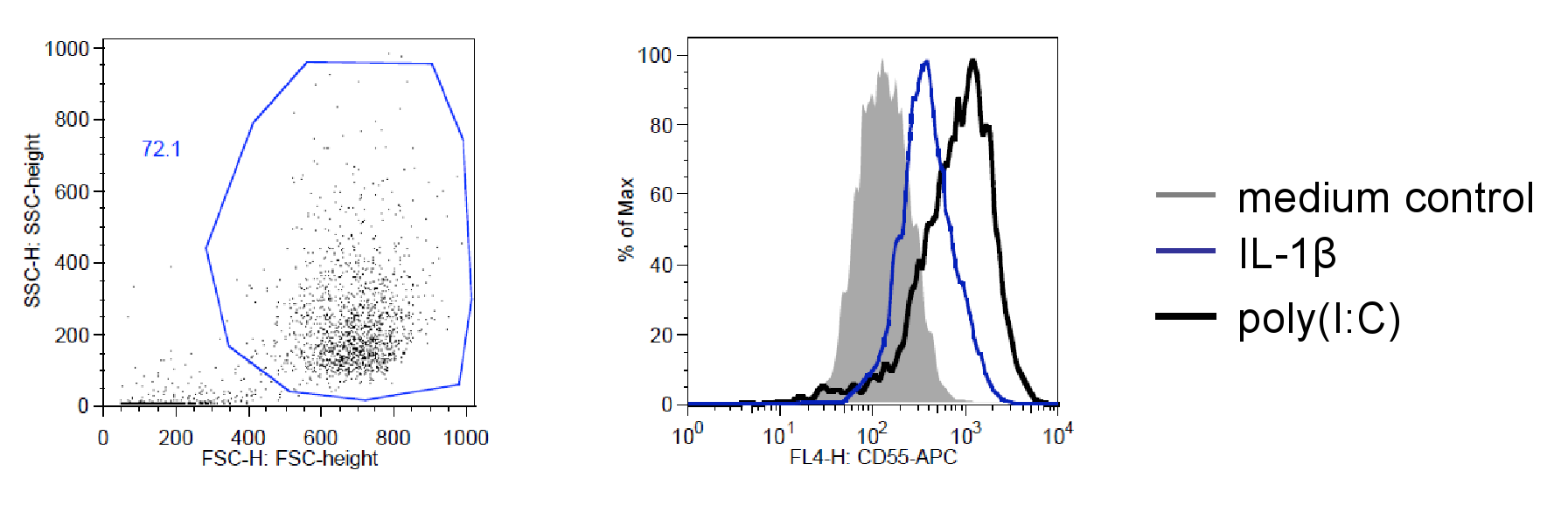

Supplement: Figure S1 — CD55 is upregulated by IL-1β and poly(I:C) on synovial fibroblasts. RA derived synovial fibroblasts were stimulated for two days with 100 ng/ml IL1β or 100 µg/ml poly(I:C). CD55 expression on synovial fibroblasts was analysed by flow cytometry. Representative scatter plot and histograms are shown. (TIF) [file pone.0035606.s001.tif]

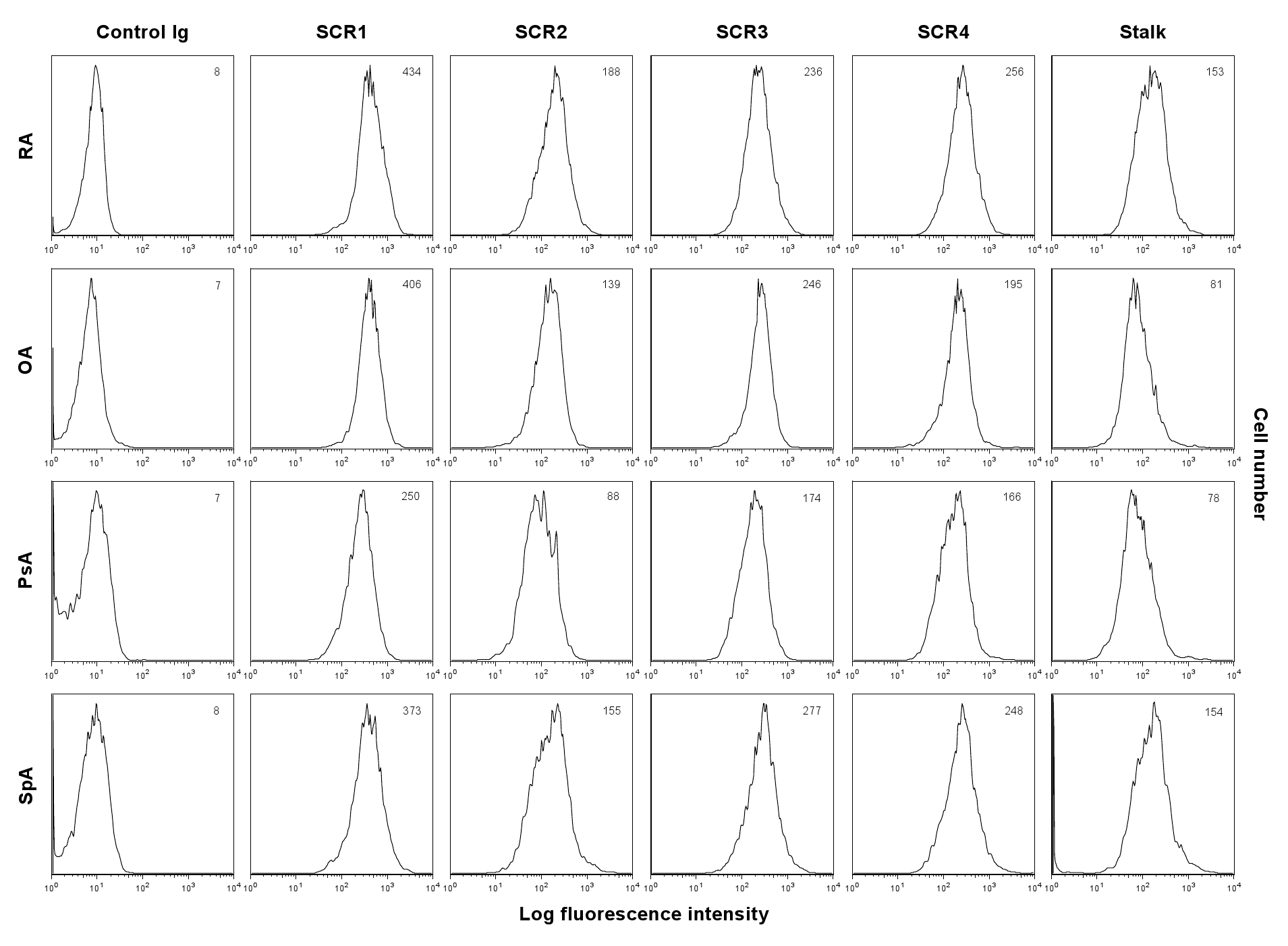

Supplement: Figure S2 — Synovial fibroblasts express full-length CD55. Synovial fibroblasts of rheumatoid arthritis (RA), osteoarthritis (OA), psoriatic arthritis (PsA), and spondyloarthritis (SpA) patients were analyzed by flow cytometry with domain-specific antibodies recognizing either the stalk or individual short consensus repeats (SCR) 1 to 4 and the stalk region of CD55. Representative histograms of 3 experiments are shown. (TIF) [file pone.0035606.s002.tif]

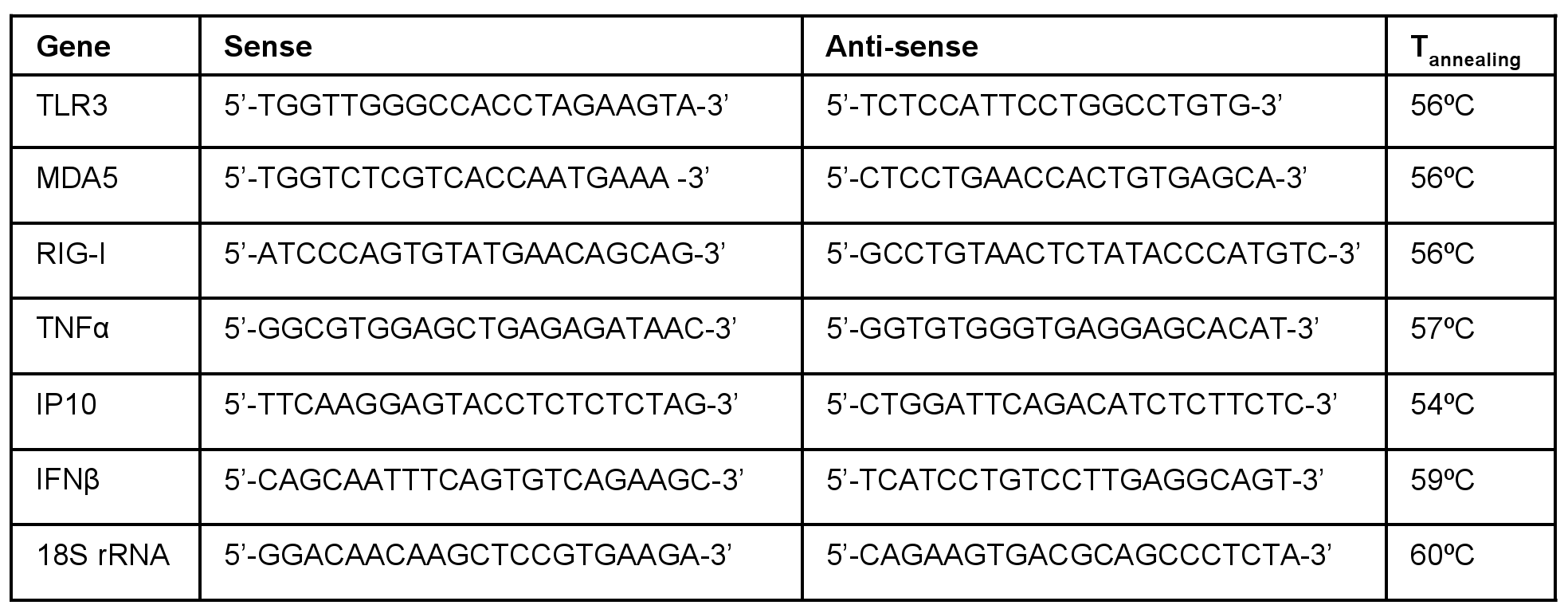

Supplement: Table S1 — PCR primer specification. (TIF) [file pone.0035606.s003.tif]
